# Validation of Anatomical Sites for the Measurement of Infrared Body Surface Temperature Variation in Response to Handling and Transport

**DOI:** 10.3390/ani9070425

**Published:** 2019-07-06

**Authors:** Luiene M. Rocha, Nicolas Devillers, Xavier Maldague, Fidèle Z. Kabemba, Julien Fleuret, Fréderic Guay, Luigi Faucitano

**Affiliations:** 1 Sherbrooke Research and Development Centre, Agriculture and Agri-Food Canada, 2000 College Street, Sherbrooke, QC J1M 0C8, Canada; 2 Département de génie électrique, Université Laval, 2325 Rue de l’Université, Quebec, QC G1V A06, Canada; 3 Département des sciences animales, Université Laval, 2425 Rue de l’Agriculture, Quebec, QC G1V 0A6, Canada

**Keywords:** behavior, handling, infrared temperature, physiological response, pigs, stress

## Abstract

**Simple Summary:**

The identification of the best anatomical site to ensure the efficient use of infrared thermography to measure body surface temperature variation in response to handling and transport stress may allow easy, non-invasive, real-time and practical animal welfare monitoring under commercial conditions. The objective of this study was to validate the anatomical sites for the measurement of infrared body surface temperature as a tool to monitor the pigs’ response to handling and transport stress. Based on the greatest variation and the closer association with heart rate and salivary cortisol found in this study, the orbital and behind ear regions (in the head) appear to be reliable points for measuring body surface temperature through the technique of thermography in response to handling and transport stress in pigs. However, based on the low to moderate correlations with other physiological indicators, infrared thermography cannot be used as a stand-alone measurement of the physiological condition of pigs in response to stress. Therefore, an appropriate use of infrared technology combined with other physiological stress indicators, e.g., heart rate, blood lactate or salivary cortisol may provide the swine industry with a tool for a real-time evaluation of the physiological condition of pigs during handling and may help to monitor critical areas during the pre-slaughter process improving animal welfare and control meat quality variation.

**Abstract:**

This study aimed at validating the anatomical sites for the measurement of infrared (IR) body surface temperature as a tool to monitor the pigs’ response to handling and transport stress. The selected anatomical sites were the neck (infrared neck temperature—IRNT), rump (infrared rump temperature—IRRT), orbital (infrared orbital temperature—IROT) and behind ears (infrared behind ears temperature—IRBET) regions. A total of 120 pigs were handled from the finishing pen to the loading dock through a handling test course. Two handling types (gentle vs. rough) and number of laps (1 vs. 3) were applied according to a 2 × 2 factorial design. After loading, pigs were transported for 40 min and returned to their home pens. Animal behavior, heart rate, rectal temperature and salivary cortisol concentration were measured for validation. Increased IR body temperature, heart rate and salivary cortisol levels were observed in response to rough handling and longer distance walk (*P* < 0.05 for all). The greatest correlations were found between IROT and IRBET temperatures and salivary cortisol concentration at the end of the handling test (*r* = 0.49 and *r* = 0.50, respectively; *P* < 0.001 for both). Therefore, IR pig’s head surface temperature may be useful for a comprehensive assessment of the physiological response to handling and transport stress.

## 1. Introduction

The stimulation of the autonomic nervous system in response to stress induces changes in peripheral vascular tone and blood flow in animals [1,2]. Indeed, when an animal becomes stressed, the hypothalamic–pituitary–adrenocortical (HPA) axis is activated because of the increase in catecholamines and cortisol concentrations, in addition to blood flow responses, and will produce changes in heat production and heat loss from the animal [3]. However, body temperature is difficult to measure under commercial conditions as the most common techniques for real-time body temperature recording, such as iButton technology [4,5,6,7,8] or radio-telemetry [9,10,11], are invasive and require animal restraint or specific training.

Thermal imaging using infrared (IR) technology is a non-invasive method for measuring skin surface temperature by converting the infrared radiation emitted by a heat source into pixel intensity [12]. Based on its rapid variation in response to acute stress [13,14], IR technology represents an alternative to invasive body temperature recording. This technology has been successfully used to assess thermal stress in cattle [15], pigs [16,17,18,19] and lambs [20], physical stress in pigs [21,22] and health conditions in cattle [23,24].

To date, the IR technology has been used to assess body surface temperature variation at either a single anatomical location, i.e., orbital, back or rump regions [21,22,25], or at multiple anatomical sites [17,19]. In previous comparative studies, the anatomical locations that allowed the most reliable assessment of body temperature and better representation of the general physiological status of pigs in response to stress were the inner ear [17], and the orbital and behind the ear-regions [19]. However, these studies only aimed at assessing the pigs’ response to thermal stress. Thus, a validation of these IR measuring sites to assess the pigs’ response to the physical stress resulting from handling and transport is needed.

Additionally, the magnitude of the correlations between IR temperature as measured at some anatomical sites (i.e., inner ear and orbital regions) and selected blood stress indicators, such as lactate and creatine kinase (CK), obtained so far only ranges from low (*r* = 0.20) to moderate (*r* = 0.55) leading to unclear conclusions [17,22]. Possible reasons for these results can be the small range of variation in stress conditions, the poor reliability of the anatomical site chosen for the IR temperature assessment and the insufficient accuracy of the IR camera used in these studies.

Therefore, the objectives of this study were two-fold: (1) To determine whether infrared body surface temperature can be used to monitor the response of pigs to handling and transport stress; and (2) to identify the anatomical site providing the most reliable validation of infrared body surface temperature as animal welfare monitoring tool in comparison with other physiological stress indicators.

## 2. Materials and Methods

All experimental procedures performed in this study followed the Canadian Council on Animal Care guidelines for humane animal use [26] and were approved by the Agriculture and Agri-Food Canada (AAFC) Animal Care Committee in Sherbrooke (473/02-10-2015/215) (QC).

### 2.1. Animals and Treatments

A total of 120 (Fertilis 25 × G-Performer 8.0, Geneticporc Inc., Saint-Bernard, Quebec, QC, Canada) commercial crossbred pigs (111.2 ± 7.8 kg live weight) were housed in individual pens (2.40 m^2^ per pig) at the Agriculture and Agri-Food Canada experimental finishing swine unit in Sherbrooke (QC, Canada) from February to March 2015.

Before the experiment, pigs were randomly identified with a numbered plastic ear tag for their recognition during the experiment and distributed into four treatments groups of 30 pigs each according to a 2 × 2 factorial design applied in a handling course. The treatments were (1) gentle handling (GH) combined with 1 or 3 laps of the handling course (GH1 and GH3, respectively), (2) rough handling (RH) combined with 1 or 3 laps of the handling course (RH1 and RH3, respectively). The distance walked by pigs doing 1 or 3 laps was 30 and 90 m, respectively. In the GH treatment, pigs were moved at a steady natural pace without being exposed to sudden movements, and loud noises or any physical contact with handler through rattle paddle and/or board (unless it was necessary, i.e., rattle paddle and plastic board) was only used to gently touch pigs when they refused to move forward, or attempted to turn back. In contrast, in the RH treatment, pigs were pushed with continuous kindly physical contact with the handler using a rattle paddle and/or plastic board to encourage pigs to walk at a fast pace.

The handling test and transportation were carried out over six days (20 pigs/day). Before each handling test, pigs were withdrawn of feed for 12 h to make them more fit for transport.

**Figure 1:**
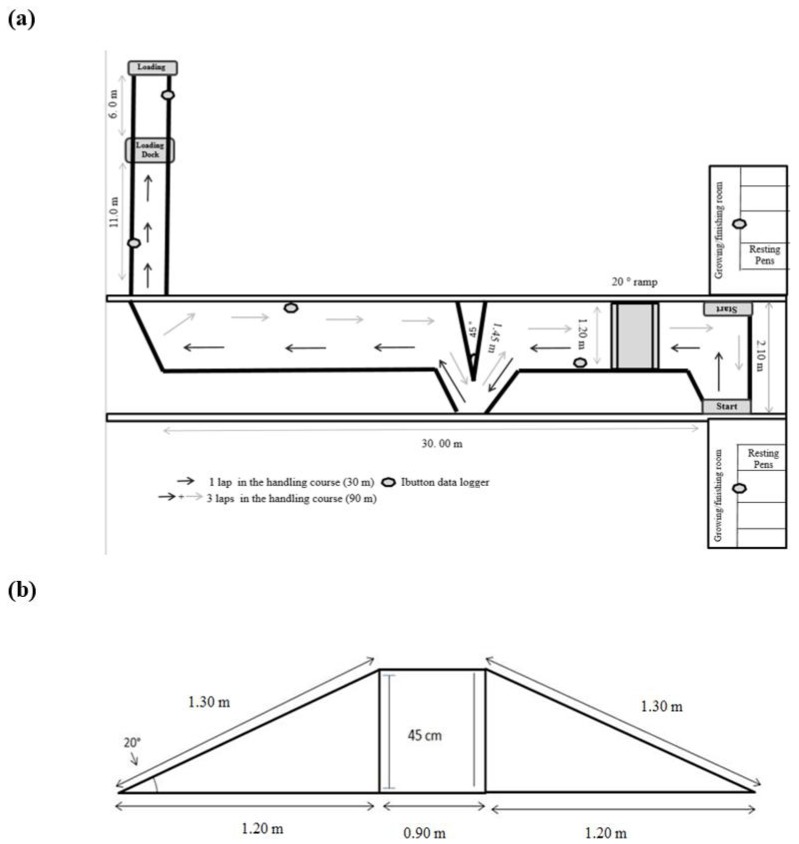
(**a**) Handling stress test course and (**b**) ramp design.

In the morning of the day of the handling test, at 08:00, pigs were handled in four treatment cohorts of five pigs each through the handling test course, starting from the finishing room exit and ending at the entrance into the loading dock. The course consisted of a 120 cm wide aisle with a 45° bend and two 90° corners (Figure 1a). Moreover, in order to increase the pigs’ physical activity and emotional stress, a 20° sloped ramp was added to the course (Figure 1b).

**Figure 2:**
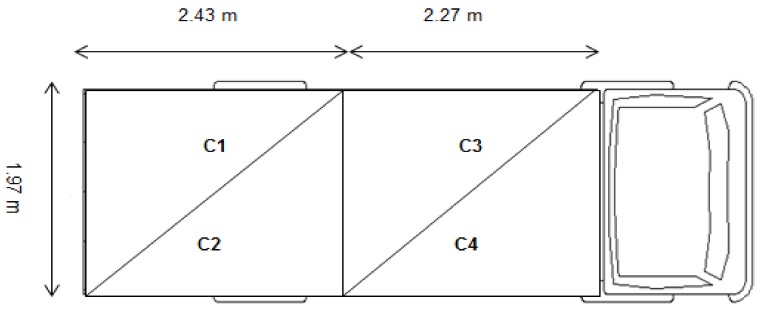
The location of compartments in the truck. (**C1**) compartment 1; (**C2**) compartment 2; (**C3**) compartment 3; (**C4**) compartment 4.

At the end of the handling test, pigs were handled through a 17 m alley to the loading dock and loaded onto a single-decked trailer, in which they were transported in four compartments separated by treatment (five pigs/compartment) for 40 min at a stocking density of 0.46 m^2^/pig (Figure 2). The treatment groups were rotated across transport compartments on each journey (six) to avoid the confounding effect of the loading order and compartment position on the pigs’ response to transport stress.

### 2.2. Ambient Condition Settings

In the finishing room, the environmental temperature was set at 20 °C (range from 18.5 to 21.5 °C during the experiment) to minimize the confounding effects of ambient temperature on IR body temperature variation. The recorded average relative humidity was 41.2% (range from 30.6% to 55.0% during the experiment). To standardize records, six iButton data loggers (DS1923 Hygrochron Temperature/Relative Humidity Logger, Maxim Integrated Products Inc., Sunnyvale, CA, USA) were distributed throughout the experimental area: Two loggers in the middle of the finishing room (overhead at a height of 1.80 m), two loggers along the handling test course (overhead at a height of 2.6 m), and another two loggers in the loading/unloading dock area (overhead at a height of 2.6 m; Figure 1a). An additional five data loggers were suspended from the ceiling of the trailer and two were hooked to the external mirrors to monitor ambient conditions within and outside the trailer, respectively, during transport. The data loggers were programmed to record the room air temperature and relative humidity at 1 min intervals within a T ° range of −20 to +85°C with an accuracy and resolution of ± 0.5 °C, and an RH range of 0% to 100% with a resolution of ± 0.6%. Data from each data loggers were downloaded after each handling and transport trial using the OneWireViewer software (Maxim Integrated Products Inc., Sunnyvale, CA, USA).

### 2.3. Infrared Thermography Measurement

Two IR thermography cameras (IR-TCM 384, 120 mm lens, Jenoptik, Jena, Germany) within an operating T° range of −25 °C to +55 °C with a measurement accuracy of ±2.0% and with a resolution of 640 × 380 pixels for images were used in this study. One camera was connected to a laptop (W530, Toshiba, Markham, ON, Canada) and manually operated by a trained technician at approximately 1.50 m perpendicular to the pig body, as recommended by Loughmiller et al. [27], to take IR images of each pig at rest (basal level) inside the finishing pen on the day before the handling test and right after the pigs’ return to the home pen after transport. The other IR camera was installed in the loading alley at a height of 2.6 m above the animals to capture IR images on each pig during loading and unloading.

Changes in the camera to object distance can substantially affect detection of the emissivity and the IR-measured temperature [25], therefore, to decrease the random variance in the IR temperature data, the distance of the IR camera in relation to the animals was considered during the interpretation of thermographic images in the IRT Cronista^®^ software.

As heat emissivity for live pigs varies among the different anatomical regions [28], the IR camera was set up at an emissivity of 0.95 which is the average value calculated on the range of 0.93 to 0.98 reported in the literature for biological tissue surface temperature [21,28,29,30].

One iButton data logger (DS1923 Hygrochron Temperature/Relative Humidity Logger, Maxim Integrated Products Inc., Sunnyvale, CA, USA) was hooked onto the IR camera to correct the camera temperature and humidity readings in the thermogram according to the ambient temperature, as recommended by Yahav and Giloh [31].

### 2.4. Capture of Infrared Thermography Images

**Figure 3:**
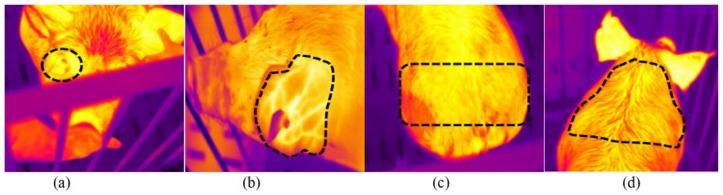
Anatomical sites used for the infrared thermography assessment: (**a**) Orbital region (infrared orbital temperature—IROT); (**b**) behind ears (infrared behind ears temperature—IRBET); (**c**) rump (infrared rump temperature—IRRT; and (**d**) neck (infrared neck temperature—IRNT).

During the four assessment periods (rest in the pen, loading, unloading and return in the pen), a total of 60 IR thermography images/s were taken on each pig body at four different anatomical sites, namely neck (infrared neck temperature—IRNT), rump (infrared rump temperature—IRRT), orbital region (infrared orbital temperature—IROT) and behind ears (infrared behind ears temperature—IRBET). The IR thermograms were analyzed using the IRT Cronista Professional software (version 3.6; Grayess, Bradenton, FL, USA) to determine the maximum, minimum and average temperature at each anatomical site of each pig in each experimental period. Each anatomical site was assessed by means of a delineated area using shapes or free-drawing tools in the IRT Cronista software (Figure 3).

### 2.5. Other Physiological Measurements

#### 2.5.1. Heart Rate

Each pig was fitted around the chest with a heart rate monitor (Team Polar, Polar Electro Canada, Lachine, QC, Canada) within an operating T° range of −10 °C to +45 °C with a measurement accuracy of 1 bpm, which was programmed to record heart rate at 5 s intervals at rest (1 h before the start of the handling test), during loading, transportation, unloading and upon the return to the home pen. For the protection and stable positioning, heart rate monitors were covered by nylon weight-lifting belts buckled around the pig’s chest and wrapped with a plastic bandage. Heart rate monitors were installed on each pig on the day before the handling test to allow them to recover from the stress of restraint and handling and avoid any bias related to this procedure on the physiological changes during the experiment. The heart rate monitors were removed immediately after the pigs returned to their home pen. Data were downloaded, and the average heart rate for each pig was determined for each of the experimental events (i.e., at rest, loading, transportation, unloading, return to home pen); the values were expressed as beats per minute (bpm).

#### 2.5.2. Rectal Temperature

Rectal temperature was assessed in pigs at rest (on the day before test, after the IR scan) and after their return to the home pen using a digital thermometer (Formedica^®^, Model 8086, Montreal, QC, Canada) within an operating T° range of 32 °C to 42.9 °C and with a measurement accuracy of 0.1 °C. The digital thermometer was placed at a sufficient depth in the rectum (approx. 10 cm) to ensure contact between the probe and the mucous lining, as suggested by Soerensen and Pedersen [14]. An average delta temperature (ΔT) value was calculated by subtracting the basal temperature (at rest) from the rectal temperature recorded upon each pig’s return to the home pen.

#### 2.5.3. Salivary Cortisol

Saliva samples were collected by a trained technician positioned outside the pen when the pigs were at rest, i.e., in the pen on the day before the experiment (between 08:30 and 09:30), and immediately after the pigs were returned to the home pen after transport (between 08:30 and 09:30) following the procedure described by [32]. Briefly, saliva samples were collected using cotton wool attached to the ends of a 1 m long plastic rod by means of rubber stoppers placed at both ends of the rod. Pigs chewed on the rubber stoppers freely for two consecutive minutes. If the cotton was not completely soaked with saliva, pigs were allowed to chew for one additional minute. To avoid the dilution of saliva, due to drinking water (which could interfere with the accuracy of the analysis), water was withdrawn for 1 h before saliva collection on the day before the handling test, and for 1 h before the handling test and transportation. After sample collection, the cotton wool was placed in plastic bags and kept refrigerated on ice for up to 30 min before being transferred to a cooler (4 °C) where it was stored for up to 1 h before manual extraction of the saliva samples by hand pressure. Saliva samples were then transferred into 1.5 mL Eppendorf tubes and stored at −80 °C pending analysis.

Salivary cortisol levels were measured in duplicate using a commercially available enzyme immunoassay kit (Salivary Cortisol EIA Kit, 1-3002 (5PK 1-3002-5), Salimetrics Inc., State College, PA, USA). The quantitative determination of salivary cortisol was performed with a microplate reader (SpectraMax Plus 384, Molecular Devices, LLC, Sunnyvale, CA, USA), and the values were expressed as ng/mL. The intra- and inter-assay coefficient of variation for salivary cortisol analysis were, respectively, 4.6% and 4.5%.

### 2.6. Behavior Measurements

For recognition, during the behavioral observations, each pig was marked with a painted number on its back. Behavior observations were performed on each group of five pigs using five digital camcorders (Panasonic WV-CP 480, Panasonic, Mississauga, ON, Canada) installed on camera mounts placed overhead along the handling test course. Images were captured and recorded with the Omnicast system (version 4.0; Genetec Inc., Montreal, QC, Canada) at a rate of 15 images/s. Two additional digital camcorders (Canon Elura 90, Canon, Ottawa, ON, Canada) were installed on overhead camera mounts on the loading/unloading ramp, and operated at a rate of 30 images/s. For each group of pigs, all occurrences of pig behavior and handler interventions needed to move the pigs through the alley were counted (Table 1). Observations started as the first pig crossed the start gate at the finishing room exit and stopped when the last pig of the group crossed the trailer gate. The total time taken to move pigs through the handling test course was also noted.

Two additional digital camcorders (DCR-HC48, Sony of Canada Ltd., Toronto, ON, Canada) were installed on camera mounts on the right front and on the left rear corners inside the trailer to observe behaviors during transport using scan-sampling at 1 min intervals (Table 1).

### 2.7. Statistical Analysis

All statistical procedures, including data and residual distribution tests, were carried out by the SAS software [35] using the animal as the experimental unit for the analysis of physiological data and the group as the experimental unit for the behavior observations. Salivary cortisol, rectal temperature and heart rate data were analyzed using the MIXED procedure of SAS in a 2 × 2 factorial arrangement with the replicate (six trial days) considered as a blocking factor in a generalized randomized complete block design (five observations per treatment combination per block), with handling type, number of laps and interaction between the two treatments included in the model. Whereas, maximum IR data were analyzed using the same model in repeated measures, including the event, i.e., sampling point (rest, handling in the course, loading, transport, unloading and return to the home pen) and the interaction between the event and anatomical sites. The IR data analysis also included a thermogram average for the maximum temperature at each body site, and ΔT values were calculated by subtracting the maximum IR basal temperature (at rest) from the maximum IR temperature recorded during each experimental period for each pig to provide the variation in skin temperature between events. Multiple comparisons of means for rectal temperature and heart rate among events were adjusted using Tukey correction.

Data are presented as least square means ± SEM. Due to frequent interference caused by the ears partially or completely hiding pig’s eyes, reliable IROT data were only obtained in 17% of pigs at loading; these data were thus excluded from the analysis for the loading experimental period.

The behavioral observations for the GH1 group on day 6 were not included in the statistical analysis, due to the poor quality of the data, i.e., extreme values for some of the behaviors observed. The frequency of animal behavior and handler interventions were analyzed with the GLIMMIX procedure in SAS using a randomized complete block design. Either logistic analysis (binary distribution with logit link function for overlap and squeeze behaviors), Poisson regression (Poisson distribution with logarithmic link function for vocalizations, back-up and slip behaviors) or negative binomial regression (negative binomial distribution with logarithmic link function for physical contact and reluctance to move behaviors) were used depending on the distribution of the variables. The postures of pigs during transportation were assessed using the MIXED procedure of SAS in repeated measures. Standing and sitting behavior data were submitted to an angular transformation to achieve normality. All behavior results correspond to the adjusted least square mean values back-transformed on the scale of original measurements with confidence limits (lower and upper limits).

The CORR procedure of SAS was used to calculate Spearman correlation coefficients between physiological measurements (e.g., rectal temperature, heart rate, and salivary cortisol) and maximum IR temperatures recorded at each anatomical site (i.e., IRNT, IRRT, IROT and IRBET), in order to determine whether IR body surface temperature measured at the different anatomical sites can be used to monitor pig welfare during handling and after transport. The Spearman correlation coefficients were also used to identify the anatomical site that would allow a more reliable IR temperature measurement and best represents the physiological response of pigs to handling and transport stress.

A probability level of *P* ≤ 0.05 was chosen as the limit for statistical significance in all tests.

## 3. Results and Discussion

### 3.1. Variation of Infrared Body Surface Temperature by Treatment and Event

As the treatment × anatomical site interaction had no effect on IR body surface temperature (*P* > 0.05) in this study, data were pooled across anatomical sites, and only the combined effects of handling type and number of laps by the event are presented and discussed (Table 2).

A greater IR maximum body surface temperature was observed in GH pigs than in RH pigs at loading (32.52 ± 0.24 vs. 30.98 ± 0.24 °C; *P* < 0.001), whereas at unloading, the RH group showed greater IR maximum body surface temperature compared to GH pigs (33.83 ± 0.25 vs. 33.09 ± 0.25 °C; *P* = 0.04). The increased IR body surface temperature observed in GH pigs during loading may result from their psychological response to the novelties of the handling test course, since the gentle handling applied to this group allowed them to explore the novel environment and to react more to it compared to RH pigs (see later section on behavior results). Whereas, IR body surface temperature variation observed in RH pigs likely resulted from their fatigue caused by the additive effect of the handling intensity during loading and transport stress, as reported in a number of previous studies [36,37].

Except for IR body surface temperature recorded on pigs’ return to the home pen, the interaction handling type × number of laps had no effect on IR body surface temperature at any time in this study (Table 2). On the return to the home pen, the IR body surface temperature of RH3 pigs was greater (+2.24 °C; *P* = 0.02) than that of RH1 pigs. This result may be explained by the additive effect of the greater handling intensity and longer walking distance on the thermal condition of RH3 pigs. The increased IR body surface temperature in the pigs likely resulted from the greater heat produced by skeletal muscle activity [38,39,40,41,42], cutaneous vasodilatation [43], and short-term exchange in the convective delivery of central heat to the periphery [44].

### 3.2. Variation of Infrared Body Surface Temperature by Anatomical Site and Event

As shown in Table 3, IRNT and IRBET progressively increased from rest to loading and from loading to unloading (*P* < 0.001 for both). In this study, IROT also increased from rest to unloading, but dropped on the return of pigs to the home pen (*P* < 0.001). Whereas, IRRT increased from the rest to loading, dropped at unloading and increased again upon the return of pigs to the home pen (*P* < 0.001). The temperature variation observed on the pig’s head may result from the effects of physical stress of loading and unloading on body temperature, as reported in previous studies [36,37], and the greater heat dissipation through blood circulation from the head [4]. The lower temperature recorded in the rump region at unloading may be explained by the reduced heat dissipation, due to the insulation provided by the thicker fat cover in this region [45].

As shown in Table 3, among the anatomical sites, the greatest variation in IR skin surface temperature between rest and unloading was observed in the orbital region (IROT ΔT = +7.01 ± 0.29 °C; *P* < 0.001) and behind the ears (IRBET ΔT = +5.86 ± 0.46 °C; *P* < 0.001). The second largest variation between the rest and the return to the home pen IR values was recorded in the neck region (IRNT ΔT = +5.73 ± 0.30 °C; *P* < 0.001) and behind the ears (IRBET ΔT = +5.67 ± 0.33 °C; *P* < 0.001). Compared with IRNT, the lower variation in IRBET values may be due to the lower skin and subcutaneous fat thickness in the region behind the ears compared with the neck regions [45]. Whereas, the greater hair density on the neck and rump surface compared to the orbital region and the ears [45] may be a factor contributing to lower variation in IR temperature in these anatomical sites. A dense, dry hair coat, in fact, increases the emissivity of the skin area, because of the cavity effect [46,47].

In this study, the highest IR temperature was recorded behind the ears (IRBET) and in the orbital (IROT) regions at unloading (*P* < 0.05; Table 3)—which may be explained by the close proximity of the eyes and ears to the brain [48], and their richer capillary beds innervated by the sympathetic system quickly responding to changes in the blood flow in stressful situations [23,49], the greater vasomotor response in the extremities than in the middle body regions [50], and the greater heat dissipation in the region of the head [16,18,19,51].

### 3.3. Heart Rate

**Figure 4:**
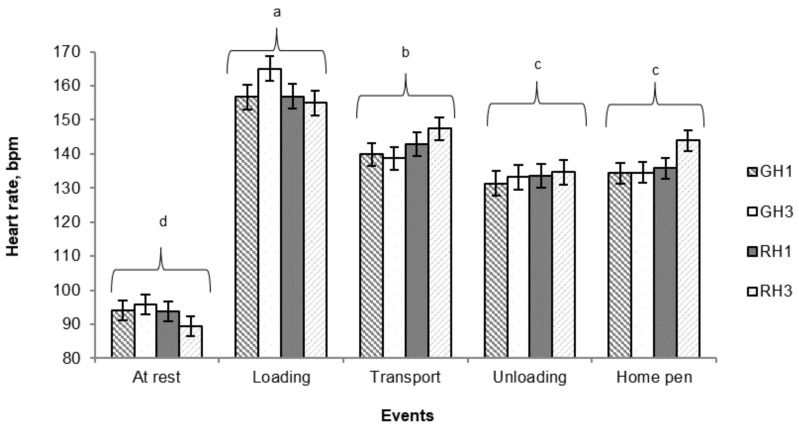
Least square means (± SEM) of heart rate variation measured at rest, during loading, during transport, during unloading and on the return to the home pen. Least square means without a common superscript differ (Tukey’s test; *P* < 0.001). GH1: One lap of the handling course with gentle handling; RH1: One lap of the handling course with rough handling; GH3: Three laps of the handling course with gentle handling; RH3: Three laps of the handling course with rough handling.

Overall, in this study, pigs’ heart rate varied between events (*P* < 0.001; Figure 4), with the rate is the highest at loading (158 ± 1.56 bpm) and dropping during transport (142.4 ± 1.7 bpm), at unloading (133.0 ± 1.7 bpm) and up to the return at the home pen (137.0 ± 1.5 bpm). However, no significant change in heart rate was found in pigs between unloading and the return to the home pen (*P* > 0.05).

As there was no interaction between handling type and number of laps, heart rate data were pooled across treatments (Table 4). Heart rate was only affected by handling type, with RH pigs showing a greater (*P* = 0.04) heart rate on their return to the home pen compared with GH pigs. No significant effect of the number of laps on pigs’ heart rate was observed in this study (*P* > 0.05).

In agreement with a number of previous reports [37,52,53,54,55], in this study, heart rates increased at loading and decreased during transport and at unloading. However, in this study, this effect was exacerbated by the additive effects of rough handling through an alley characterized by multiple corners and a sloped ramp. Increased heart rates have been previously reported in pigs handled with electric prods [53], negotiating closed corners or bends [56] and sloped ramps [57,58,59] at loading.

### 3.4. Variation of Rectal Temperature by Event

No significant effect of the handling type or number of laps, either as a single factor or interaction, was found on rectal temperature during the events (*P* > 0.05; Table 5). This result is difficult to explain, since rectal temperature has been commonly used as an indicator of stress in several studies [60,61,62]. In this study the lack of effect of the handling intensity or the distance moved on the rectal temperature may be related to the time lapse between unloading, driving pigs back to the home pen and rectal temperature measurement (approx. 10–15 min.). However, other studies also failed to report an effect of handling intensity or distance moved on the rectal temperature variation in pigs [60,63]. Additionally, it cannot be ruled out that the size differences between animals used in this study and those reported studies may be an important factor for the lack of results.

**Figure 5:**
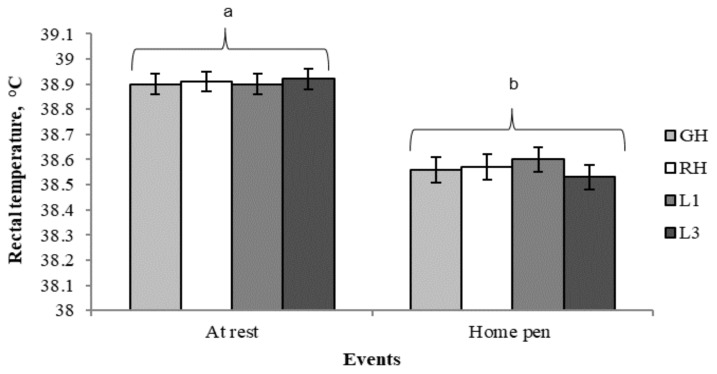
Least square means (± SEM) of rectal temperature measured at rest and on the return to the home pen. Least square means without a common superscript differ (Tukey’s test; *P* < 0.001). GH: Gentle handling; RH: Rough handling; L1: One lap of the handling course; L3: Three laps of the handling course.

Therefore, data were pooled across treatments to show the variation of rectal temperature between rest and the end of the handling/transport test (Figure 5). The average rectal temperature was slightly greater at rest than on pigs’ return to the home pen (38.9 ± 0.03 °C vs. 38.6 ± 0.03 °C; *P* < 0.001), however, both values are within the reference range reported for slaughter pigs (39.3 ± 0.03 °C; [14]). Additionally, the higher rectal temperature at rest may result from the relatively high basal blood flowing from hotter visceral structures to the rectum, making it, along with the gastrointestinal tract, the body site with the highest core temperature [64].

### 3.5. Salivary Cortisol

Overall, in this study, salivary cortisol level increased from rest (basal level) to the pigs’ return to their home pen (1.84 ± 0.07 to 3.30 ± 0.10; *P* < 0.001). As shown in Table 6, the interaction handling type × number of laps had an impact on salivary cortisol levels in this study, with greater (*P* < 0.001) cortisol levels being found in RH3 pigs compared with RH1 ones on the return to the home pen. This increase in salivary cortisol may result from the additive effect of emotional and physical stress experienced by pigs that had to walk a greater distance at a fast pace. These results are in agreement with those reported by Cook et al. [65] who also found increased salivary cortisol concentrations resulting from the rise in the adrenocortical activity in response to handling and transport stress.

### 3.6. Behavioral Response

In this study, GH pigs took longer to load than the RH pigs (4.03 ± 0.53 vs. 1.47 ± 0.05 min; *P* < 0.001). Correa et al. [53] also reported a shorter loading time for pigs handled harshly (i.e., electric prodding) compared with those driven gently (i.e., with paddles) as they are imposed to walk at a fast pace. As expected, in this study driving pigs through three laps instead of only one lap of the handling test course took longer (3.71 ± 0.47 vs. 1.79 ± 0.26 min; *P* < 0.01).

As there was no interaction between handling type and number of laps on loading time and pig behaviors, data were pooled across treatments (Table 7).

Similar to Correa et al. [53] and Rabaste et al. [66], in this study rough handling resulted in more pigs overlapping compared with more gentle handling (*P* = 0.05; Table 7). However, when compared with RH pigs, GH pigs backed-up, turned back and vocalized more, and were more reluctant to move (*P* < 0.001). These behaviors may indicate that when gentle handling is applied, pigs are more inclined to walk at a pace that allows them to explore the novel environment and express fear behaviors, such as reluctance to move and turning-back [67], towards the novelties encountered through the handling test course (i.e., ramp, bends and sharp corners in this study). Goumon et al. [58] also reported a greater frequency of fear behaviors in near-market-weight pigs being handled through ramps or corners.

When compared to one lap, imposing pigs to walk three laps through the handling test course increased the frequency of physical contacts from the handler (*P* = 0.05), reluctance to move (*P* = 0.04) and turns-back (*P* < 0.001). The physical effort to go through a longer walking distance and to go up and down a ramp three times may elicit increased frustration and fatigue in this group of pigs, leading to the higher incidence of those fear behaviors, and the need of more handler interventions to move forward. Previous studies [68,69,70] also observed signs of physical signs of stress during loading in pigs moved over a long distance compared with those moved over a short one.

Overall in this study, pigs only stood and sat during transport, regardless of handling type or number of laps (*P* > 0.05; Table 8). These postures are normally observed in the truck during trips as short as that of this study [34,71,72]. Pigs, in fact, start to accommodate in the truck and lie down only between 0.5 and 3 h from the departure from the farm, regardless of the loading conditions [73,74,75].

### 3.7. Correlations between IR Temperature at Different Anatomical Sites, Other Physiological Stress Indicators and Behavior Measurements

Spearman correlation coefficients between IRNT, IRRT, IROT and IRBET, and physiological measurements are shown in Table 9. Among the anatomical sites, the greatest correlation was found between IROT and IRBET (*r* = 0.90; *P* < 0.001), followed by that between IROT and IRNT (*r* = 0.84; *P* < 0.001) and IRNT and IRBET (*r* = 0.82; *P* < 0.001). Previous studies also reported strong correlations between IR temperature values at these anatomical sites in pigs [19,51].

The greater magnitude of the correlation between IROT and IRBET may be explained by the greater heat dissipation through blood circulation from the head [44]. Additionally, according to Ng et al. [76], the accuracy of surface temperature measurements at the orbital and ears locations is even better compared with other anatomical regions, due to the lower fat thickness and hair coat density reducing the distance between the blood flow and the body skin in these regions. This difference in measurement accuracy between anatomical sites was also observed in our study, where low correlations were observed between IROT or IRBET and IRNT regions.

In this study, all correlations between physiological stress indicators and IR temperature values at different anatomical sites were significant (*P* < 0.001), except for IRNT and heart rate (*r* = 0.13; *P* > 0.05), but were from weak to moderate (*r* = −0.27 to 0.56). The greatest correlation was found between IROT and heart rate (*r* = 00.56; *P* < 0.001), which is not surprising as heart rate and IR ocular temperature are both indicators of physical stress [22,58]. Greater correlations were also found between IR temperatures at all anatomical sites and salivary cortisol (*r* = 0.49–0.51; *P* < 0.001). Similar or higher correlations (*r* = 0.55 to 0.71) between the IR maximum ocular temperature and salivary cortisol have also been reported in horses [77,78]. However, our results are in disagreement with those reported by Warriss et al. [17] who failed to find a relationship between IR ear temperature and serum cortisol level. The discrepancy in the results between the two studies may be explained by the greater responsiveness of salivary cortisol to stress-related adrenocortical activity compared to plasma cortisol [77]. Therefore, based on its association with salivary cortisol variation, it may be concluded that the variation in the IR body surface temperature—especially that assessed in the head region, may also be influenced by the hypothalamic-pituitary-adrenal axis activity in response to the physical stress or exercise during handling and transport in pigs, leading to an alteration in heat production and loss from the animal [3].

Significant, although rather weak, the correlation was found between IROT and rectal temperature (*r* =−0.32; *P* < 0.001). This result suggests that a greater variation in IROT during physical effort may be explained by the different dissipation rate between core, i.e., rectal temperature, and superficial temperatures (i.e., orbital IR skin surface temperature) resulting from the difference in blood flow redistribution during physical effort. In this study, IROT temperature increased from rest to loading (29.05 ± 0.26 °C vs. 36.06 ± 0.26 °C), while rectal temperature (38.9 ± 0.03 °C vs. 38.6 ± 0.03 °C) remained stable. Indeed, this result indicates that during physical effort the sympathetic nervous system is activated and catecholamines are released into the bloodstream resulting in vasodilation and increased blood flow in the region of the head [79]. As the blood flow changes in response to physical activity, the amount of radiated heat that is lost from the orbital region is increased, whereas the parasympathetic system reduces gastrointestinal activity by decreasing blood flow towards the intestinal tract [80], likely caused by the vasoconstriction of the rectal wall in contrast to the orbital region [81].

No significant correlations between IR body surface temperature at different anatomical sites and behavioral measurements were found in this study (*P* > 0.05).

## 4. Conclusions

In this study, the body surface temperature, as assessed by infrared thermography varied according to the anatomical location chosen for this measurement. However, based on their greatest variation and their closer association with heart rate and salivary cortisol found in this study, the orbital and behind ear regions appear to be the most recommendable anatomical location for the infrared temperature assessment in response to handling and transport stress in pigs. Nevertheless, given the moderate level of correlation between the infrared temperatures assessed at these anatomical sites and other physiological stress indicators, the infrared thermography cannot be recommended as a stand-alone measurement of the physiological condition of pigs in response to stress. Additionally, measuring body IR temperature in the orbital region may be difficult under commercial conditions because of the frequent movement of the head, making the IR thermal scan imprecise. More research is needed to refine the accuracy of the infrared technology, to determine the time lapse for the increase in IR body surface temperature and to make the method more adaptable for use in commercial settings. 

## Figures and Tables

**Table 1:** Ethogram of handler behavior toward pigs and pig behavior during loading and transport (adapted from References [33,34]).

Observation	Description
**Handler behavior**	
Physical intervention	Handler uses a paddle or a board (at loading) to gently push and encourage the forward movement of one pig or a group of pigs
**Pig behavior**	
During handling	
Slip/fall	Pig’s leg splits away from the other legs or pig falls down (at least two legs buckled under)
Overlap	Pig mounts another pig, with its two front legs on the back of the other pig
Turn-back	Pig makes a 180° turn, ending with its rear extended in the direction of intended movement
Back-up	Pig moves at least two steps rearward, opposite the direction of intended motion
Vocalization	Pig vocalizes
Reluctance to move	Pig refuses to walk or stops for more than 2 s
During transport	
Standing	Pig upright, with no part of the torso in contact with the floor
Sitting	Pig upright, with the hindquarters in contact with the floor
Lying	Pig with the full torso in contact with the floor

**Table 2:** Least square means of infrared body surface temperature by treatment and event.

	Treatment ^1^					*P* Value ^2^		
Event	GH1	GH3	RH1	RH3	SEM	Handling	Laps	Handling × Laps
Loading	32.26	32.78	30.89	31.08	0.35	< 0.001	ns	ns
Unloading	33.10	33.08	33.46	34.20	0.36	0.04	ns	ns
Home pen	34.00	35.11	33.78	36.02	0.24	ns	< 0.001	0.02

^1^ GH1: One lap of the handling course with gentle handling; RH1: One lap of the handling course with rough handling; GH3: Three laps of the handling course with gentle handling; RH3: Three laps of the handling course with rough handling. ^2^ ns: Non-significant (*P* > 0.05).

**Table 3:** Least square means of infrared body surface temperature by anatomical site and event.

	Event				*P* Values	
Anatomical site ^1^	At rest	Loading	Unloading	Home pen	SEM	Event
IRNT	28.42 ^c^	29.73 ^b^	33.77 ^a^	34.15 ^a^	0.47	<0.001
IRRT	29.18 ^b^	34.02 ^a^	28.56 ^b^	34.45 ^a^	0.50	<0.001
IROT	29.05 ^c^	^**^	36.06 ^a^	34.32 ^b^	0.26	<0.001
IRBET	29.60 ^c^	33.51 ^b^	35.44 ^a^	35.25 ^a^	0.45	<0.001

^1^ IRNT: Infrared neck temperature; IRRT: Infrared rump temperature; IROT: Infrared orbital temperature; IRBET: Infrared behind ears temperature Within a row, means with different superscripts differ (Tukey’s test; *P* < 0.05). ^**^ Missing data.

**Table 4:** Least square means of heart rate (bpm) by treatment and event.

	Handling Type ^1^				Number of Laps ^1^			
Event	GH	RH	SEM	*P* value ^2^	1	3	SEM	*P* value ^2^
Loading	160.8	155.6	2.00	ns	156.5	159.9	2.03	ns
Transport	139.3	145.0	2.38	ns	141.0	143.3	2.38	ns
Unloading	132.1	133.8	2.41	ns	132.1	133.8	2.41	ns
Home pen	134.2	139.7	1.90	0.04	134.9	139.0	1.89	ns

^1^ GH: Gentle handling; RH: Rough handling; L1: One lap of the handling course; L3: Three laps of the handling course ^2^ ns: Non-significant (*P* > 0.05).

**Table 5:** Least square means of rectal temperature by treatment and event.

	Treatment ^1^					*P* Value ^2^		
Event	GH1	GH3	RH1	RH3	SEM	Handling	Laps	Handling × Laps
At rest	38.91	38.89	38.87	38.96	0.06	ns	ns	ns
Home pen	38.63	38.48	38.57	38.57	0.07	ns	ns	ns

^1^ GH1: One lap of the handling course with gentle handling; RH1: One lap of the handling course with rough handling; GH3: Three laps of the handling course with gentle handling; RH3: Three laps of the handling course with rough handling ^2^ ns: Non-significant (*P* > 0.05).

**Table 6:** Least square means of salivary cortisol level (ng/mL) by treatment and event.

	Treatments ^1^					*P* values ^2^		
Event	GH1	GH3	RH1	RH3	SEM	Handling	Laps	Handling × Laps
At rest	1.68	1.77	1.75	2.11	0.14	ns	ns	ns
Home pen	3.36	2.90	2.95	4.09	0.21	ns	ns	< 0.001

^1^ GH1: One lap of the handling course with gentle handling; RH1: One lap of the handling course with rough handling; GH3: Three laps of the handling course with gentle handling; RH3: Three laps of the handling course with rough handling ^2^ ns: Non-significant (*P* > 0.05).

**Table 7:** Back-transformed least squares means (confidence limits) of behaviors observed during the handling test and at loading according to the handling type and number of laps in the handling course.

	Handling Type ^1^			Number of Laps ^1^		
Behavior	GH	RH	*P* value ^2^	1	3	*P* value ^2^
Overlap	0.13	0.60	0.05	0.27	0.37	ns
	(0.01–0.57)	(0.18–0.91)		(0.04–0.75)	(0.07–0.82)	
Slip	0.50	0.61	ns	0.33	0.76	ns
	(0.21–0.79)	(0.26–0.87)		(0.12–0.65)	(0.41–0.93)	
Back-up	5.44	1.67	< 0.001	2.37	3.85	ns
	(3.41–8.60)	(0.91–3.09)		(1.32–4.25)	(2.35–6.32)	
Reluctance to move	28.07	1.54	< 0.001	4.36	9.89	0.04
	(18.6–42.3)	(0.79–2.98)		(2.35–8.09)	(6.15–15.9)	
Turn-back	2.24	0.48	< 0.001	0.47	2.24	< 0.001
	(1.18–4.24)	(0.18–1.26)		(0.18–1.26)	(1.18–4.23)	
Physical contact	2.34	57.07	< 0.001	9.02	14.82	0.05
	(1.48–3.71)	(47.2–69.0)		(6.19–13.1)	(10.7–20.6)	
Vocalization	3.07	0.33	< 0.001	0.85	1.20	ns
	(2.13–4.40)	(0.11–0.97)		(0.38–1.90)	(0.55–2.63)	

^1^ GH: Gentle handling; RH: Rough handling; ^2^ ns: Non-significant (*P* > 0.05).

**Table 8:** Back-transformed least squares means (confidence limits) of postures observed during transport according to the handling type and number of laps in the handling course.

	Treatments ^1^				*P* Values ^2^
Postures	GH1	GH3	RH1	RH3	
Standing, %	96.95	99.10	95.50	97.20	ns
	(91.15–99.80)	(95.20–100)	(88.85–99.24)	(91.50–99.82)	
Sitting, %	2.22	0.46	3.70	2.03	ns
	(0.06–7.40)	(0.00–3.70)	(0.46–9.82)	(0.03-7.05)	

^1^ GH1: One lap of the handling course with gentle handling; RH1: One lap of the handling course with rough handling; GH3: Three laps of the handling course with gentle handling; RH3: Three laps of the handling course with rough handling ^2^ ns: Non-significant (*P* > 0.05).

**Table 9:** Spearman correlations between infrared body surface temperatures assessed at four different anatomical sites and physiological indicators.

Anatomical Site ^1^	IRNT	IRRT	IROT	IRBET	Rectal T °	Heart Rate	Salivary Cortisol
IRNT	1.00	0.48 ^***^	0.84 ^***^	0.82 ^***^	−0.27 ^*^	0.13	0.49 ^***^
							
IRRT		1.00	0.41 ^***^	0.53 ^***^	−0.31 ^***^	0.34^***^	0.51 ^***^
							
IROT			1.00	0.90 ^***^	−0.32 ^***^	0.56^***^	0.50 ^***^
							
IRBET				1.00	−0.30 ^***^	0.29^*^	0.49 ^***^

^1^ IRNT: Infrared neck temperature; IRRT: Infrared rump temperature; IROT: Infrared orbital temperature; IRBET: Infrared behind ears temperature ^*^ *P* < 0.05; ^***^ *P* < 0.001.
